# Analysis of the relationship between axial length, optic disc morphology, and regional variations in retinal vessel density in young adults with healthy eyes

**DOI:** 10.3389/fmed.2023.1280048

**Published:** 2024-01-04

**Authors:** Yanhui Chen, Hua Rong, Yuling Liu, Huijuan Gao, Ziwen Sun, Weiyu Dang, Kunpeng Lu, Baoyue Mi, Jing Li, Ruihua Wei

**Affiliations:** ^1^Tianjin Key Laboratory of Retinal Functions and Diseases, Tianjin Branch of National Clinical Research Center for Ocular Disease, Eye Institute and School of Optometry, Tianjin Medical University Eye Hospital, Tianjin, China; ^2^Tangshan Ophthalmic Hospital, Tangshan, Hebei, China

**Keywords:** optic disc tilt, optic disc rotation, myopia, parapapillary retinal vessel, lens position

## Abstract

**Purpose:**

To investigate the relationship between optic disc morphology, axial length, and regional distribution of retinal vessels in healthy eyes of young adults.

**Methods:**

Nine hundred and two healthy eyes were enrolled in this university-based, cross-sectional study. Spectral-domain optical coherence tomography angiography was used to measure the parapapillary retinal vessel density. We automated the process of calculating optic disc tilt and rotation by using a program written in Python. Relationships between optic disc rotation, optic disc tilt, parapapillary vessel density, and other ocular parameters were analyzed using regression models.

**Results:**

As axial length increased, optic disc morphology became more tilted and rotated inferiorly. The superficial vessel density (SVD) and radial peripapillary capillary density (RPCD) gradually decreased in all regions except for the temporal quadrant. Increased temporal SVD (OR [95% CI] = 1.081 [1.039, 1.124], *p* < 0.001), reduced nasal SVD (OR [95% CI] = 0.898 [0.861, 0.937], *p* < 0.01), and short relative lens position (OR [95% CI] = 0.126 [0.032, 0.495], *p* = 0.003) were significantly associated with the presence of a tilted optic disc. Inferior disc rotation was associated with decreased superior deep vessel density (DVD) and increased inferior DVD and temporal DVD after adjusting for sex and axial length.

**Conclusion:**

The tilted and rotated optic discs were associated with the distribution of SVD and DVD, respectively. We should fully consider the influence of optic disc morphology on parapapillary vessel density in eyes with myopia.

## Introduction

Myopic eyes, especially highly myopic eyes, are often associated with changes in the morphology of the optic disc and in the density of retinal vessels, which are closely related to increased susceptibility to glaucoma (1–8). The increasing prevalence of myopia and glaucoma worldwide has been recognized (6). Optic disc rotation and tilt are essential in these ocular diseases. Several studies have focused on the relationship between optic disc morphology and axial length (AL) to investigate the mechanism linking myopia and glaucoma (1–5, 7, 8). The direction of optic disc rotation and tilt corresponds to the location of retinal nerve fiber layer (RNFL) defects in primary open-angle glaucoma (9). However, distinguishing glaucomatous changes from myopia is often difficult due to the confounding optic nerve head (ONH) morphology (10). In addition, there are large errors in the manual measurement of optic disc tilt or the assessment of optic disc morphology.

AL elongation also leads to changes in the circumpapillary microvasculature. The superficial retinal vessel density decreases, and a loss of microvasculature in the deep retinal layer is experienced, which is associated with glaucoma (11–13). Rotation and tilt of the optic disc represent oblique insertions of the nerve fiber bundle into the lamina cribrosa (14), which may lead to specific axon damage owing to microvascular dysfunction and intraocular pressure (IOP)-related strain on the respective laminal cribrosa pore path.

In the progression of myopia, visual cues drive the connective tissue remodeling mechanisms, and the changes in the ONH and microvasculature remain relatively stable (15, 16). Under certain pathological conditions, such as elevated IOP and hemodynamic changes, rotation, and tilt, the ONH suffers from increased susceptibility to glaucoma. Previous studies have found that myopia-related ONH morphology is correlated with myopia-related functional damage. Still, few studies have explored the effect of ONH morphology on the distribution of parapapillary vessels in healthy myopia (1–5, 7–9). In this study, we automated the process of calculating optic disc tilt and rotation by using a program written in Python, which can save considerable time and effort compared to manual calculations. We aimed to provide updated data to assess the relationship between ONH morphology and the regional distribution of parapapillary retinal vessels. These results will help to clarify the characteristics of parapapillary vessels and may contribute to the understanding of the pathogenesis of glaucoma in myopia.

## Methods

This university-based, cross-sectional study enrolled first-year students attending Tianjin Medical University in September 2020. This study adhered to the tenets of the Declaration of Helsinki and was approved by the Ethics Committee of Tianjin Medical University Eye Hospital, Tianjin, China.

### Subject inclusion

All subjects underwent a complete ophthalmic examination that included best-corrected visual acuity and slit-lamp biomicroscopy. IOP was measured with iCare Pro (Tiolat Oy, Helsinki, Finland). IOL Master 700 (Carl Zeiss Meditec, Jena, Germany) was used to measure ocular biometric parameters, including AL, central corneal thickness (CCT), anterior chamber depth (ACD), and lens thickness (LT). Relative lens position (RLP) was assessed using the following formulas: RLP = [ACD + 1/2LT]/AL × 10. Systolic and diastolic blood pressures were measured using an automated electronic blood pressure monitor. Mean ocular perfusion pressure (MOPP) = 2/3 mean blood pressure – IOP. Mean blood pressure = diastolic blood pressure + 1/3 (systolic blood pressure − diastolic blood pressure).

Spectral-domain optical coherence tomography angiography (SD-OCTA, Canon OCT-HS100, Tokyo, Japan) examinations were performed to evaluate retinal vessel density: radial peripapillary capillary density (RPCD), deep vessel density (DVD), and superficial vessel density (SVD).

#### Inclusion criteria

(1) Healthy students aged between 17 and 20; (2) AL of between 21.0 mm and 29 mm; (3) astigmatism within ±3.00 D, best corrected visual acuity of 20/25 or better; (4) IOP of 21 mmHg or lower, normal ONH without glaucomatous changes.

#### Exclusion criteria

(1) Pathologic myopic fundus changes, such as posterior staphyloma, lacquer cracks, patchy atrophy, neovascularization, or traction retinopathy; (2) ocular diseases other than myopia; (3) a history of intraocular or refractive surgery or trauma; systemic diseases that may affect ocular or cerebral blood supply (e.g., hypertension, hypotension, diabetes, migraine, and Cowden syndrome + Lhermitte Duclos disease); (4) poor OCTA image with a signal strength of 6 or lower, a motion artifact score of 3 or 4, or segmentation errors.

### OCTA imaging

SD-OCTA was used for parapapillary retinal vessel imaging. This particular system had an optical resolution of 5 μm in the axial direction and 20 μm in the transverse direction and a scanning frequency of 70,000 A-scans per second. The built-in SD-OCTA software (Ophthalmic Software Platform RX, V4.5) offers a “denoised OCTA image” that is assisted by deep learning, which shows excellent potential for automated data analysis and improved image quality (17).

OCTA images were acquired with a cube scan protocol that covered a 6 × 6 mm^2^ area centered on the ONH (464 A-scans × 464 B-scans). This automatically segmented the retina into radial peripapillary capillary, superficial, and deep layers. The radial peripapillary capillary layer extended from the internal limiting membrane to the RNFL; the superficial layer spread from the internal limiting membrane to the inner plexiform layer; and the deep layer extended from the inner plexiform layer to the outer plexiform layer. Retinal vessel density in a given region, defined as the percentage area occupied by vessels, was automatically calculated in an ETDRS circle within a 3-mm-diameter band in a different layer and manually corrected for segmentation. Parapapillary vessel density was measured in the temporal, superior, nasal, and inferior regions, excluding the central 1-mm diameter circle, which was manually centered on the optic disc.

### Tilt ratio and rotation degree measurements

**Figure 1:**
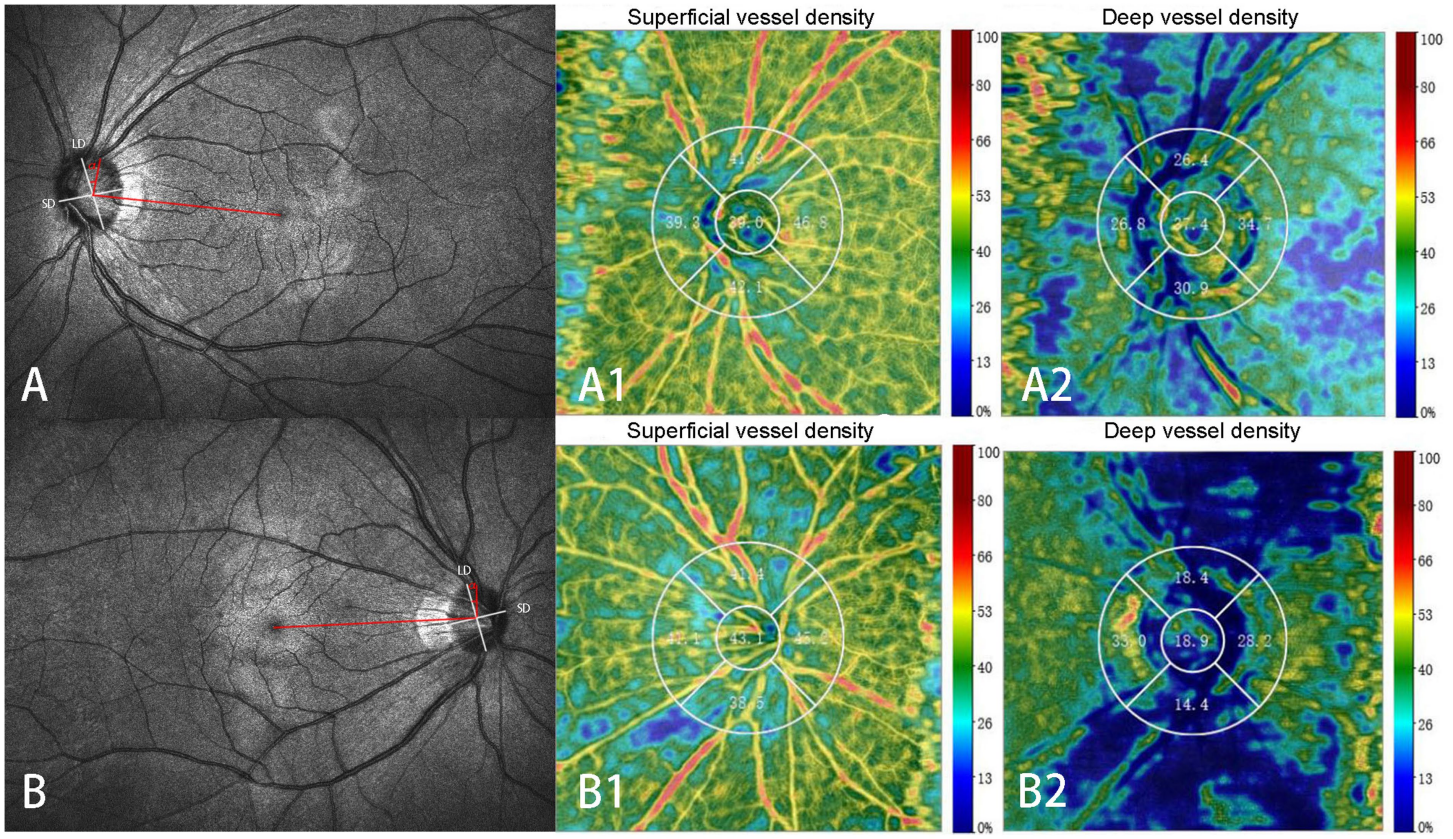
Identification of the optic disc tilt ratio and degree of rotation. The ratio of the shortest diameter (SD) to the longest diameter (LD) of the optic disc is defined as the tilt ratio. The angle (α) between the LD (white lines) and the horizontal line connecting the fovea and the center of the optic disc (red lines) is defined as the degree of rotation. The negative value indicates the LD rotated nasally **(A)**, and the positive value indicates the LD rotated temporally **(B)**. Superficial **(A1, B1)** and deep **(A2, B2)** retinal vessel densities at different regions are displayed in **A** and **B**. Superficial capillary: Superficial retinal vessel densities **(A1, B1)**; Deep capillary: retinal vessel densities **(A2, B2)**.

The tilt ratio of optic discs has defined the value of the minimum-to-maximum disc diameter. An optic disc with a tilt ratio of 0.80 or less was considered to be tilted. The degree of optic disc rotation was defined as the degree of deviation of the maximum axial deviation from the reference line, 90° from a horizontal line connecting the fovea and the center of the optic disc (where the maximum and minimum disc diameters intersect). Superior rotation, presented as a negative value, indicated the longest optic axis rotated nasally, and inferior rotation, presented as a positive value, indicated the longest optic axis rotated temporally (Figure 1) (7, 18). The optic disc was classified as having significant rotation when the degree of rotation exceeded 15° (7, 18).

Scanning laser ophthalmoscopy was performed simultaneously, and there was complete co-localization with B-scan OCT findings during OCTA imaging. The optic disc border (inner margin of the scleral ring) and the fovea were identified on B-scans and scanning laser ophthalmoscopy images and were contoured using Photoshop software on exported images (13). The tilt ratio and degree of rotation of the optic disc were calculated using a program written in Python (version 3.7.9), which can save considerable time and effort compared to manual calculations.

All eyes were classified into four groups based on AL: AL1 (21.0–24.0 mm), AL2 (24.1–25.0 mm), AL3 (25.1–26.0 mm), and AL4 (26.1–28.5 mm). Eyes were divided into a tilted optic disc group (with a tilt ratio of 0.8 or lower) and a non-tilted optic disc group (with a tilt ratio greater than 0.8). Eyes were further divided into an inferior rotation group (degree of rotation greater than 15°), a superior rotation group (degree of rotation lower than-15°), and a non-rotation group (degree of rotation between-15° and 15°).

### Statistical analyses

The normality of the data was assessed using the Kolmogorov–Smirnov test. Differences in continuous variables were compared using a *t*-test or one-way analysis of variance test with Bonferroni’s *post-hoc* test, and categorical variables were compared using the Chi-squared test. Logistic regression analyses were used to identify factors associated with a tilted optic disc. Stepwise multiple regression analyses were used to investigate the factors related to the degree of optic disc rotation. We used AL and SVD instead of the spherical equivalent and RPCD in the multivariate regression analysis. Because axial myopia is related to the ocular morphology and was not affected by the status of the crystalline lens, there was collinearity between the RPCD and the SVD. All variables with a significance level of less than 0.10 in the univariate regression model were included in the multivariate model. Statistical analyses were performed using SPSS Statistics 25.0 (IBM Corp.). Data are expressed as means ± standard deviations, or ratios. Differences were considered statistically significant when *p* < 0.05.

## Results

### General characteristics

Nine hundred and two eyes of 451 subjects initially met the inclusion criteria. Subsequently, 11 eyes were excluded because of poor-quality OCTA images, and 20 eyes were excluded because of pathologic myopia. Thus, this analysis included 871 eyes.

**Figure 2:**
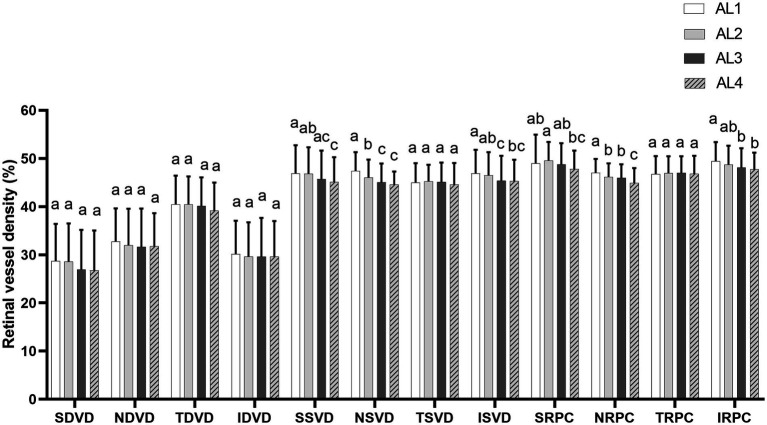
The average level of parapapillary vessel density in four peripapillary regions (superior, inferior, nasal, and temporal quadrants) among four groups (AL1, AL2, AL3, and AL4). Superficial vessel density and radial peripapillary capillary density became thinner as AL increased in the superior, inferior, and nasal quadrants (all *p* < 0.05). No significant differences were found between the four groups. ^a,b,c^Statistical differences between groups are indicated by letters, with groups sharing at least one similar letter presenting no statistically significant difference (*p* ≥ 0.05) and groups with all different letters presenting a significant difference (*p* < 0.05). SDVD, superior deep vessel density; NDVD, nasal deep vessel density; TDVD, temporal deep vessel density; IDVD, inferior deep vessel density; SSVD, superior superficial vessel density; NSVD, nasal superficial vessel density; TSVD, temporal superficial vessel density; ISVD, inferior superficial vessel density; SRPC, superior radial peripapillary capillary density; NRPC, nasal radial peripapillary capillary density; TRPC, temporal radial peripapillary capillary density; IRPC, inferior radial peripapillary capillary density.

**Figure 3:**
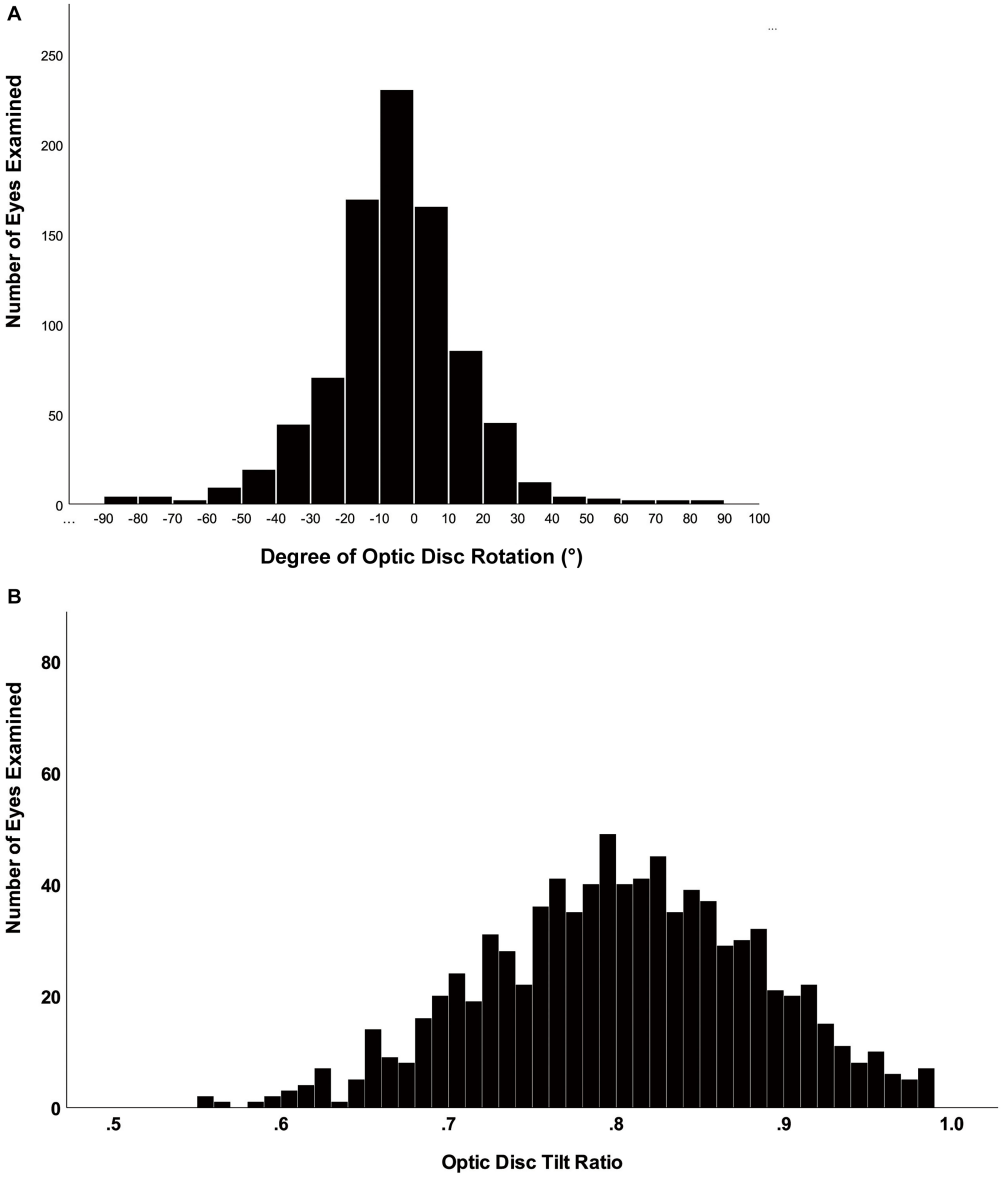
Histogram showing the distribution of the tilt ratio and degree of rotation of the optic disc in healthy eyes. **(A)** The distribution of optic disc rotation degree was normal. **(B)** The distribution of the optic disc tilt ratio was fairly normal, although right-skewed.

For all eyes, the general characteristics and regional distribution of retinal vessel density among AL1, AL2, AL3, and AL4 groups are shown in Table 1 and Figure 2. The AL4 group had the smallest degree of optic disc rotation (−3.08 ± 18.8°) and optic disc tilt ratio (0.79 ± 0.08) compared with other groups. The SVD and RPCD became thinner as AL increased in the superior, inferior, and nasal quadrants (all *p* < 0.05). No significant difference was found in DVD in the four groups. The distributions of the optic disc rotation degree and the tilt ratio were reasonably normal (Figure 3).

**Table 1:** Demographic and ocular characteristics of the study population.

	All (*n* = 871)	AL1 (*n* = 132)	AL2 (*n* = 257)	AL3 (*n* = 297)	AL4 (*n* = 185)	*p*	*Post-hoc* test (group)
Age, year	18.26 ± 0.63	18.24 ± 0.64	18.29 ± 0.71	18.28 ± 0.63	18.19 ± 0.51	0.406^†^	/
Gender, frequency of female subjects (%)	563 (64.6)	108 (81.8)	181 (70.4)	174 (58.6)	100 (54.1)	**<0.001** ^‡^	1 = 2; 1>3; 1>4; 2>3; 2>4; 3 = 4
Disc rotation°	−5.48 ± 20.06	−8.4 ± 23.48	−8.17 ± 17.72	−3.35 ± 20.72	−3.08 ± 18.8	**0.004** ^†^	1 = 2; 1<3; 1<4; 2<3; 2<4; 3 = 4
Disc tilt ratio	0.8 ± 0.08	0.83 ± 0.08	0.81 ± 0.08	0.8 ± 0.08	0.79 ± 0.08	**<0.001** ^†^	1>2; 1>3; 1>4; 2 = 3; 2 = 4; 3 = 4
AL, mm	25.16 ± 1.1	23.43 ± 0.55	24.57 ± 0.29	25.53 ± 0.28	26.62 ± 0.5	**<0.001** ^†^	1<2; 1<3; 1<4; 2<3; 2<4; 3<4
CCT, μm	539.92 ± 33.54	537.6 ± 34.34	538.77 ± 37.62	540.09 ± 31.31	542.88 ± 30.33	0.499^†^	/
ACD, mm	3.68 ± 0.26	3.45 ± 0.24	3.64 ± 0.23	3.75 ± 0.23	3.78 ± 0.23	**<0.001** ^†^	1<2; 1<3; 1<4; 2<3; 2<4; 3 = 4
LT, mm	3.46 ± 0.18	3.55 ± 0.17	3.48 ± 0.17	3.44 ± 0.18	3.42 ± 0.17	**<0.001** ^†^	1>2; 1>3; 1>4; 2 = 3; 2>4; 3 = 4
RLP, mm	2.15 ± 0.09	2.23 ± 0.09	2.19 ± 0.08	2.14 ± 0.08	2.06 ± 0.09	**<0.001** ^†^	1>2; 1>3; 1>4; 2>3; 2>4; 3>4
SE, D	−4.22 ± 2.42	−1.6 ± 1.52	−3.23 ± 1.67	−4.77 ± 1.95	−6.6 ± 1.86	**<0.001** ^†^	1>2; 1>3; 1>4; 2>3; 2>4; 3>4
Including eyes, percentage of the right eye (%)	469 (53.8)	61 (46.2)	147 (57.2)	153 (51.5)	108 (58.4)	0.092^‡^	/
IOP, mmHg	15.52 ± 1.79	15.65 ± 1.81	15.33 ± 1.87	15.51 ± 1.90	15.69 ± 1.45	0.147^†^	/
S-DVD, %	27.65 ± 8.09	28.74 ± 7.67	28.58 ± 7.91	26.95 ± 8.2	26.73 ± 8.31	0.016^†^	1 = 2 = 3 = 4
N-DVD, %	31.93 ± 7.47	32.75 ± 6.88	31.97 ± 7.6	31.63 ± 7.98	31.77 ± 6.86	0.542^†^	/
T-DVD, %	40.06 ± 5.9	40.45 ± 5.98	40.44 ± 5.82	40.11 ± 5.96	39.19 ± 5.8	0.128^†^	/
I-DVD, %	29.69 ± 7.46	30.13 ± 6.93	29.62 ± 7.12	29.6 ± 8.03	29.61 ± 7.37	0.909^†^	/
S-SVD, %	46.06 ± 5.68	46.9 ± 5.83	46.78 ± 5.52	45.67 ± 5.95	45.09 ± 5.17	**0.003** ^†^	1>4; 2>4; 1 = 2; 1 = 3; 2 = 3; 3 = 4
N-SVD, %	45.58 ± 3.76	47.37 ± 3.91	45.99 ± 3.78	45.03 ± 3.92	44.6 ± 2.68	**<0.001** ^†^	1>2; 1>3; 1>4; 2>3; 2>4; 3 = 4
T-SVD, %	45.02 ± 3.95	45 ± 4.01	45.23 ± 3.44	45.11 ± 4.02	44.6 ± 4.43	0.391^†^	/
I-SVD, %	45.92 ± 4.91	46.89 ± 4.89	46.48 ± 4.81	45.37 ± 5.18	45.31 ± 4.41	**0.002** ^†^	1 = 2; 1>3; 1>4; 2>3; 2 = 4; 3 = 4
S-RPC, %	48.82 ± 4.46	48.97 ± 6	49.54 ± 3.88	48.75 ± 4.41	47.81 ± 3.8	**0.001** ^†^	1 = 2; 1 = 3; 1 = 4; 2 = 3; 2>4; 3 = 4
N-RPC, %	45.95 ± 2.96	47.01 ± 2.88	46.16 ± 2.83	45.95 ± 2.82	44.89 ± 3.1	**<0.0001** ^†^	1>2; 1>3; 1>4; 2 = 3; 2>4; 3>4
T-RPC, %	46.9 ± 3.57	46.74 ± 3.75	46.93 ± 3.52	47 ± 3.46	46.83 ± 3.73	0.900^†^	/
I-RPC, %	48.4 ± 3.91	49.43 ± 3.94	48.71 ± 3.92	48.08 ± 4.04	47.74 ± 3.46	**<0.001** ^†^	1 = 2; 1>3; 1>4; 2 = 3; 2 = 4; 3 = 4
MOPP, mmHg	41.86 ± 4.54	41.48 ± 4.88	41.97 ± 4.58	42.20 ± 4.31	41.42 ± 4.56	0.219	/

Factors with statistical significance are shown in bold. ACD, anterior chamber depth; LT, lens thickness; RLP, relative lens position; SE, spherical equivalent; IOP, intraocular pressure; S-DVD, superior deep vessel density; N-DVD, nasal deep vessel density; T-DVD, temporal deep vessel density; I-DVD, inferior deep vessel density; S-SVD, superior superficial vessel density; N-SVD, nasal superficial vessel density; T-SVD, temporal superficial vessel density; I-SVD, inferior superficial vessel density; S-RPC, superior radial peripapillary capillary density; N-RPC, nasal radial peripapillary capillary density; T-RPC, temporal radial peripapillary capillary density; I-RPC, inferior radial peripapillary capillary density; MOPP, mean ocular perfusion pressure. ^†^One-way analysis of variance test. ^‡^*χ*^2^ test.

### Characteristic assessment of subjects with and without optic disc tilt

The difference between the tilted disc and the non-tilted disc is shown in Table 2. Among the 871 eyes, 431 (49.5%) belonged to the non-tilted disc group, and 440 (50.5%) belonged to the tilted disc group. Compared with the non-tilted group, the tilted group had a smaller degree of rotation, a longer AL, a larger refractive error, and a shorter RLP. More women than men had tilted optic discs. No significant difference was found for AD, LT, or IOP.

**Table 2:** Comparison of characteristics of subjects with and without optic disc tilt.

	Non-tilted disc (*n* = 431)	Tilted disc (*n* = 440)	*p*
Age, year	18.26 ± 0.62	18.25 ± 0.65	0.776^†^
Gender, frequency of female subjects (%)	264 (61.3)	299 (68.3)	**0.039** ^‡^
Disc rotation°	−6.95 ± 24.51	−4.04 ± 14.31	**0.032** ^†^
Disc tilt ratio	0.87 ± 0.04	0.74 ± 0.05	**<0.001** ^†^
AL, mm	25.03 ± 1.11	25.29 ± 1.07	**<0.001** ^†^
CCT, μm	540.55 ± 34.85	539.30 ± 32.24	0.583^†^
ACD, mm	3.68 ± 0.27	3.68 ± 0.24	0.682^†^
LT, mm	3.46 ± 0.18	3.46 ± 0.17	0.848^†^
RLP, mm	2.17 ± 0.11	2.14 ± 0.09	**<0.001** ^†^
SE, D	−3.89 ± 2.45	−4.55 ± 2.34	**<0.001** ^†^
IOP, mmHg	15.58 ± 1.86	15.45 ± 1.72	0.286^†^
S-DVD, %	28.33 ± 8.24	26.99 ± 7.89	**0.014** ^†^
N-DVD, %	32.39 ± 7.52	31.48 ± 7.41	0.074^†^
T-DVD, %	39.99 ± 6.06	40.14 ± 5.75	0.697^†^
I-DVD, %	29.71 ± 7.68	29.67 ± 7.24	0.946^†^
S-SVD, %	46.26 ± 5.64	45.86 ± 5.72	0.306^†^
N-SVD, %	46.11 ± 3.76	45.05 ± 3.68	**<0.001** ^†^
T-SVD, %	44.75 ± 3.68	45.28 ± 4.18	**0.047** ^†^
I-SVD, %	45.97 ± 5.04	45.86 ± 4.78	0.757^†^
S-RPC, %	48.98 ± 4.20	48.66 ± 4.71	0.285^†^
N-RPC, %	46.30 ± 2.78	45.60 ± 3.09	**0.001** ^†^
T-RPC, %	46.64 ± 3.38	47.16 ± 3.74	**0.032** ^†^
I-RPC, %	48.38 ± 3.92	48.41 ± 3.90	0.929^†^
MOPP, mmHg	41.83 ± 4.43	41.88 ± 4.65	0.864

Factors with statistical significance are shown in bold. ACD, anterior chamber depth; LT, lens thickness; RLP, relative lens position; SE, spherical equivalent; IOP, intraocular pressure; S-DVD, superior deep vessel density; N-DVD, nasal deep vessel density; T-DVD, temporal deep vessel density; I-DVD, inferior deep vessel density; S-SVD, superior superficial vessel density; N-SVD, nasal superficial vessel density; T-SVD, temporal superficial vessel density; I-SVD, inferior superficial vessel density; S-RPC, superior radial peripapillary capillary density; N-RPC, nasal radial peripapillary capillary density; T-RPC, temporal radial peripapillary capillary density; I-RPC, inferior radial peripapillary capillary density; MOPP, mean ocular perfusion pressure. ^†^Student’s *t*-test. ^‡^*χ*^2^ test.

The tilted disc group had lower superior DVD, lower nasal SVD and RPCD, and greater temporal SVD and RPCD than the non-tilted group.

### Characteristic assessment of subjects with different optic disc rotation directions

The general characteristics of the subjects with the different optic disc rotation directions are presented in Table 3. Among the 871 eyes, 546 eyes (62.7%) belonged to the non-rotation group, 97 eyes (11.1%) belonged to the inferior rotation group, and 228 eyes (26.2%) belonged to the superior rotation group. The smallest tilt ratio was found in the non-rotation group (*p* < 0.001). Compared with the superior rotation group, there was longer AL, greater refractive error, shorter RLP, higher IOP, and more women in the inferior rotation group. No significant difference was found in CCT, AD, LT, age, or the presence of a tilted disc between the inferior rotation group and the superior rotation group.

**Table 3:** Comparison of characteristics of eyes with and without optic disc rotation.

	Non-rotation (*n* = 546)	Inferior rotation (*n* = 97)	Superior rotation (*n* = 228)	*p*	*Post-hoc* test (group)
Age, year	18.25 ± 0.68	18.24 ± 0.72	18.27 ± 0.62	0.896^†^	/
Gender, frequency of female subjects (%)	375/(68.7)	67 (69.1)	121 (53.1)	**<0.001** ^‡^	1>3; 2>3; 1 = 2
Optic rotation°	−1.56 ± 8.02	28.17 ± 14.62	−29.2 ± 14.54	**<0.001** ^†^	1<2; 1>3; 2>3
Disc tilt ratio	0.79 ± 0.08	0.84 ± 0.08	0.83 ± 0.08	**<0.001** ^†^	1<2; 1<3
With tilted optic disc, (%)	322 (59.0%)	33 (34.0%)	85 (37.3*%*)	**<0.001** ^‡^	1>2; 1>3; 2 = *3*
AL, mm	25.21 ± 1.06	25.31 ± 1.1	24.97 ± 1.17	**0.007** ^†^	1>3; 1 = 2; 2>3
CCT, μm	540.53 ± 33.19	533.12 ± 36.49	541.33 ± 32.89	0.102^†^	/
AD, mm	3.68 ± 0.24	3.65 ± 0.29	3.69 ± 0.27	0.506^†^	/
LT, mm	3.45 ± 0.18	3.5 ± 0.2	3.47 ± 0.17	**0.032** ^†^	1<2; 1 = 3; 2 = 3
RLP	2.15 ± 0.10	2.14 ± 0.09	2.17 ± 0.10	**0.002** ^†^	1<3; 2<3; 1 = 2
SE, D	−4.43 ± 2.37	−4.62 ± 2.22	−3.57 ± 2.48	**<0.001** ^†^	1>3; 1 = 2; 2>3
IOP, mmHg	15.30 ± 1.74	16.54 ± 1.95	15.52 ± 1.79	**<0.001** ^†^	1<2; 1 = 3; 2>3
S-DVD, %	27.79 ± 8.07	25.77 ± 8.54	28.13 ± 7.87	**0.045** ^†^	2<3; 1 = 2; 1 = 3
N-DVD, %	32.25 ± 7.3	31.04 ± 7.5	31.54 ± 7.85	0.224^†^	/
T-DVD, %	40.22 ± 5.87	40.54 ± 5.56	39.5 ± 6.09	0.215^†^	/
I-DVD, %	30 ± 7.31	31.67 ± 7.59	28.11 ± 7.48	**<0.001** ^†^	1>3; 2>3; 1 = 2
S-SVD, %	46.07 ± 5.64	45.93 ± 5.06	46.1 ± 6.04	0.968^†^	/
N-SVD, %	45.56 ± 3.54	45.13 ± 3.86	45.81 ± 4.18	0.318^†^	/
T-SVD, %	45.27 ± 4.1	45.09 ± 3.67	44.39 ± 3.62	**0.017** ^†^	2>3; 1 = 2; 1 = 3
I-SVD, %	46.04 ± 4.69	45.67 ± 4.89	45.72 ± 5.41	0.610^†^	/
S-RPC, %	48.97 ± 4.22	48.56 ± 3.63	48.56 ± 5.28	0.421^†^	/
N-RPC, %	45.84 ± 2.8	45.93 ± 3.02	46.19 ± 3.3	0.323^†^	/
T-RPC, %	47.23 ± 3.58	47.13 ± 3.02	46.03 ± 3.63	**<0.001** ^†^	1>3; 2>3; 1 = 2
I-RPC, %	48.52 ± 3.95	48.43 ± 3.52	48.1 ± 3.97	0.393^†^	/
MOPP, mmHg	41.92 ± 4.78	40.89 ± 3.87	42.11 ± 4.16	0.073	/

Factors with statistical significance are shown in bold. ACD, anterior chamber depth; LT, lens thickness; RLP, relative lens position; SE, spherical equivalent; IOP, intraocular pressure; S-DVD, superior deep vessel density; N-DVD, nasal deep vessel density; T-DVD, temporal deep vessel density; I-DVD, inferior deep vessel density; S-SVD, superior superficial vessel density; N-SVD, nasal superficial vessel density; T-SVD, temporal superficial vessel density; I-SVD, inferior superficial vessel density; S-RPC, superior radial peripapillary capillary density; N-RPC, nasal radial peripapillary capillary density; T-RPC, temporal radial peripapillary capillary density; I-RPC, inferior radial peripapillary capillary density; MOPP, mean ocular perfusion pressure. ^†^One-way analysis of variance test. ^‡^*χ*^2^ test.

The inferior rotation group had a lower superior DVD, higher inferior DVD, temporal SVD, and temporal RPCD than the superior rotation group.

### Factors associated with the presence of a tilted disc

Logistic regression analysis showed that decreased nasal SVD, increased temporal SVD, and short RLP were significantly associated with higher odds of tilted optic discs (Table 4). We found that the odds of having a tilted disc decreased by 10% for each 1% increase in nasal SVD, 87% for each 1 mm posterior shift in RLP, and increased by 8% for each 1% increase in temporal SVD.

**Table 4:** Factors associated with tilted optic discs in all subjects.

		Univariate Analysis		Multivariate analysis^*^
	*p*	OR (95% CI)	*p*	OR (95% CI)
S-DVD, %	**0.014**	0.98 (0.96, 1.00)		
N-DVD, %	0.074	0.98 (0.97, 1.00)		
T-DVD, %	0.696	1.00 (0.98, 1.03)		
I-DVD, %	0.946	1.00 (0.98, 1.02)		
S-SVD, %	0.306	0.99 (0.96, 1.01)		
N-SVD, %	**<0.001**	0.93 (0.89, 0.96)	**<0.001**	0.90 (0.86, 0.94)
T-SVD, %	**0.048**	1.04 (1.00, 1.07)	**<0.001**	1.08 (1.04, 1.12)
I-SVD, %	0.757	1.00 (0.97, 1.02)		
Gender (male subjects/female subjects)	**0.039**	0.75 (0.56, 0.99)		
AL, mm	**<0.001**	1.25 (1.10, 1.41)		
ACD, mm	0.682	0.90 (0.53, 1.51)		
RLP, mm	**<0.001**	0.08 (0.02, 0.29)	**0.003**	0.13 (0.03, 0.49)
IOP, mmHg	0.286	0.97 (0.89, 1.03)		
Disc rotation, °	**0.033**	1.01 (1.00, 1.01)		

^*^Variables with *p* < 0.1 in the univariate analysis were included in the subsequent multivariate analysis. Factors with statistical significance are shown in bold. S-DVD, superior deep vessel density; N-DVD, nasal deep vessel density; T-DVD, temporal deep vessel density; I-DVD, inferior deep vessel density; S-SVD, superior superficial vessel density; N-SVD, nasal superficial vessel density; T-SVD, temporal superficial vessel density; I-SVD, inferior superficial vessel density; ACD, anterior chamber depth; RLP, relative lens position; IOP, intraocular pressure.

### Factors associated with optic disc rotation

Multivariate linear regression results showed that the increased degree of optic disc rotation (more inferiorly rotated optic disc) was associated with decreased superior DVD, and was associated with increased inferior DVD and temporal DVD, and associated with greater AL and female. Each 1-mm increase in AL was associated with a 0.13° increase in the degree of rotation. Each 1 mmHg increase in IOP was associated with a 0.11° increase the degree of rotation. Each 1% increase in temporal DVD and inferior DVD was associated with a 0.12° increase and a 0.26° increase in the degree of rotation, respectively. Each 1% increase in superior DVD was associated with a 0.28° decrease in the degree of rotation. The degree of optic disc rotation was 0.13° lower in men than in women. The combination of these factors yielded an R2 of 0.121 and an adjusted R2 of 0.115 (Table 5).

**Table 5:** Multivariate regression analysis of associations with optic disc rotation in all subjects.

	Univariate analysis			Multivariate analysis^*^		
	β	*p*	B (95% CI)	β	*p*	B (95% CI)
S-DVD, %	−0.08	**0.016**	−0.20 (−0.37, −0.04)	−0.28	**<0.001**	−0.70(−0.91, −0.48)
N-DVD, %	0.02	0.575	0.05 (−0.13, 0.23)			
T-DVD, %	0.08	**0.017**	0.27 (0.05, 0.50)	0.12	**0.008**	0.41 (0.11, 0.71)
I-DVD, %	0.17	**<0.001**	0.46 (0.29, 0.64)	0.26	**<0.001**	0.70 (0.48, 0.93)
S-SVD, %	0.03	0.363	0.11 (−0.13, 0.34)			
N-SVD, %	−0.02	0.523	−0.12 (−0.47, 0.24)			
T-SVD, %	0.09	**0.010**	0.44 (0.11, 0.78)			
I-SVD, %	0.02	0.600	0.07 (−0.20, 0.35)			
Gender (male subjects/female subjects)	−0.11	**0.001**	−4.63 (−7.40, −1.85)	−0.13	**<0.001**	−5.46(−8.18, −2.74)
AL, mm	0.12	**<0.001**	2.19 (0.98, 3.40)	0.13	**<0.001**	2.46 (1.28, 3.64)
ACD, mm	−0.03	0.442	−2.05 (−7.28, 3.18)			
RLP, mm	−0.13	**<0.001**	−25.90 (−38.91, −12.88)			
IOP, mmHg	0.11	**0.001**	1.28 (0.54, 2.02)	0.11	**0.001**	1.22 (0.52, 1.92)
Disc tilt ratio	−0.08	**0.024**	−18.95(−35.44, −2.46)			

^*^Variables with *p* < 0.1 in the univariate analysis were included in the subsequent multivariate analysis. Factors with statistical significance are shown in bold. S-DVD, superior deep vessel density; N-DVD, nasal deep vessel density; T-DVD, temporal deep vessel density; I-DVD, inferior deep vessel density; S-SVD, superior superficial vessel density; N-SVD, nasal superficial vessel density; T-SVD, temporal superficial vessel density; I-SVD, inferior superficial vessel density; ACD, anterior chamber depth; RLP, relative lens position; IOP, intraocular pressure.

## Discussion

In the current study, we compared the differences in the optic disc morphology in healthy eyes with different AL and analyzed the relationship between the optic disc appearance, AL, and regional distribution of retinal vessel density in young adults. This research provides a perspective for differentiating glaucoma from myopia from the point of view of ONH morphology and hemodynamics.

The ONH is significantly more obliquely oriented in eyes with myopia than in eyes without myopia (19). The peripapillary scleral flange at the temporal and inferior disc border pulled the optic nerve dura mater backward, resulting in the tilted optic disc due to the displacement of Bruch’s membrane opening (8, 19–21). ONH stretching associated with axial elongation occurs asymmetrically, with a more substantial impact in the direction of the longest axis of the disc and a weaker effect in the direction of the shortest axis of the disc (19). During AL elongation, the retina becomes thinner, the vessel diameter becomes smaller, and the oxygen demand decreases, leading to a decrease in vessel density (22). We found that the optic disc morphology became more elliptical (lower optic disc tilt ratio) with increasing AL, and the SVD and RPCD gradually decreased in all regions except for the temporal quadrant.

Current results showed that a tilted disc was related to decreased nasal SVD, increased temporal SVD, and an anterior shift of the lens. The presence of optic disc tilt was not associated with DVD in any region. Due to AL elongation and optic disc tilt, the fovea moved farther from the optic disc and the retinal vascular arch became shorter (20). Therefore, the RNFL and superficial retinal vessels were dragged to the temporal quadrant, resulting in a decrease in nasal and superior vascular density (21, 23). Sung et al. (24) found that the reduction of mean superficial vessel density was related to the increase of AL and the decrease of RNFL thickness, which was not associated with the disc tilt. Our result differed from that of Sung et al., and the possible reason was that those authors analyzed the mean vessel density in the radial peripapillary capillaries and choroidal layers. Moreover, increased optic disc tilt may change the RNFL rule (25, 26), and the mean vessel density may not reflect the distribution characteristic, which may lead to an overdiagnosis of glaucoma (15).

It was found that RLP gradually moved anteriorly with the AL elongation (27). Hyperopic defocus caused by myopia-related anteriorly displaced lenses leads to vitreous chamber elongation and retinal shape change (27). Moreover, anterior lens position may be caused by the vitreous chamber elongation exceeding the increase in ACD. Our results showed that a shorter RLP was correlated with the presence of a tilted optic disc. However, the effect of anterior lens position on optic disc tilt still needs further investigation.

During AL elongation, the optic disc morphology indicated a rotational appearance in the healthy myopic eye and glaucoma (1, 3, 5, 7, 8). Although superior rotation related to myopia is more common than inferior rotation, the latter is mainly found in highly myopic glaucoma (5, 7). Inferior rotation was related to elevated IOP and thin RNFL (7). The direction of disc rotation may predict the location of visual field defects and visual field sensitivity loss (28, 29). Rotation around the sagittal axis is usually seen in highly myopic eyes, most often with rotation of the superior disc pole in the temporal direction and without any change in the disc diameter (1, 8). Our results showed that 325 eyes (59.5%) had apparent optic disc rotation, while the proportion of superior rotation was 70.2% (228 eyes). Consistent with previous studies, the direction of rotation was more inferior in eyes with AL greater than 26 mm than in other groups.

There is a significant spatial correlation between decreased retinal vessel density and decreased visual field sensitivity in myopic subjects (30). Both elevated IOP and parapapillary hemodynamic disorder were associated with glaucoma (31). In the current research, we found that the inferior rotation group had a higher IOP than the superior rotation group. The former had a lower superior DVD and a higher inferior DVD. SVD and RPC were mainly increased in the temporal quadrant. Univariate and multivariate linear regression results showed that, after adjusting for AL and sex, increased degree of rotation (inferior rotation) was related to decreased superior DVD, increased inferior and temporal DVD, and increased IOP.

Despite a significant spatial correlation between decreased retinal vessel density and decreased visual field sensitivity (30), the fovea was still slightly inferior to the disc center in an eye with a rotated disc, which means that the spatial relationship between the optic disc and the fovea remains (19). Therefore, the decrease in the superior DVD and the increase in the temporal DVD caused by the optic disc rotation may be mainly attributed to the redistribution of retinal vessels. The inferior DVD was not related to the disc rotation. The fovea located at the lower position of the ONH center resulted in a relatively flat course of the inferior nerve fiber bundle (26). When the optic disc rotates downward, the deformation of the lower axon is less significant than that of the upper one (26).

It is worth noting that optic disc rotation is related to the DVD but independent of the SVD. We speculated that the deep retinal vessels mainly come from the choroid and the short posterior ciliary artery branches. The force to induce eye rotation is essentially different from that to induce optic disc tilt (19). The drive of the internal orbital force may participate in the rotation of the optic disc (32, 33) and then influence the deep retinal vessels through the short posterior ciliary artery. Additionally, an increased degree of rotation may increase distortion and deformation of axons or tortuosity of the lamina cribrosa pore path, which has been associated with significant VF defects or lamina cribrosa changes in high myopia or glaucoma (16, 34, 35). The upper and lower edges of the lamina cribrosa lack connective tissue support; they are more sensitive to IOP changes and blood supply status. Whether the decreased superior DVD could cause obstacles to the blood supply of the upper lamina cribrosa pore path needs to be further explored.

Furthermore, studies have shown that there is a notable difference in the gender distribution among different AL groups. It was found that more women had tilted optic discs and belonged to the inferior rotation group. However, multivariate analysis revealed that gender was not associated with the presence of disc tilt, which is consistent with previous research (18). In contrast, it was observed that gender was linked to the direction of optic rotation, with women more likely to exhibit inferior rotation. This finding was partly attributed to the fact that women tend to have more optic disc dysversion at birth, owing to the gender difference in the severity of embryonic optic fissure malclosure (36).

### Limitations

First, the relationship between ONH morphology and vessels needs to be determined in a longitudinal study. Second, the recruited subjects are regular university students, and the relationship between vessel density and ONH morphology in glaucoma needs further investigation. Third, ONH morphology was assessed on two dimensions, which may not precisely reflect the actual shape of the optic disc. Three-dimensional evaluation is necessary to analyze ONH morphology and explore the relationship between rotation and tilt of the ONH. Finally, it should be noted that although the inclusion criteria for the current study were based on the absence of glaucomatous changes in the ONH, the use of ONH analysis for the diagnosis of glaucoma is still controversial. Therefore, it is possible that some subjects with glaucoma may not have been excluded as a visual field test was not conducted. However, since the participants in this study were young students with normal vision, the impact of glaucoma on the results was negligible.

## Conclusion

In conclusion, our Python software utilizes image processing techniques to automatically detect the optic disc in retinal images. Once the optic disc is identified, the software calculates its tilt and rotation angles with high accuracy. Our results indicated that the tilted and rotated discs were associated with the distribution of superficial and deep retinal vessel density, respectively. When distinguishing vascular abnormalities from myopia, we should fully consider the influence of ONH morphology on parapapillary vessel density.

## Data availability statement

The original contributions presented in the study are included in the article/supplementary material, further inquiries can be directed to the corresponding author.

## Ethics statement

The studies involving humans were approved by Ethics Committee of Tianjin Medical University Eye Hospital. The studies were conducted in accordance with the local legislation and institutional requirements. Written informed consent for participation was not required from the participants or the participants’ legal guardians/next of kin as it was a retrospective and noninvasive study without any medical intervention.

## Author contributions

YC: Conceptualization, Formal analysis, Methodology, Writing – original draft, Writing – review & editing. HR: Software, Visualization, Writing – review & editing. YL: Data curation, Investigation, Writing – review & editing. HG: Formal analysis, Visualization, Writing – review & editing. ZS: Investigation, Methodology, Writing – review & editing. WD: Investigation, Methodology, Writing – review & editing. KL: Investigation, Methodology, Writing – review & editing. BM: Methodology, Project administration, Writing – review & editing. JL: Investigation, Methodology, Writing – review & editing. RW: Conceptualization, Writing – review & editing.
